# Case report of a molar-root incisor malformation in a patient with an autoimmune lymphoproliferative syndrome

**DOI:** 10.1186/s12903-019-0739-z

**Published:** 2019-03-22

**Authors:** Alenka Pavlič, Milka Vrecl, Janja Jan, Milan Bizjak, Ana Nemec

**Affiliations:** 1 Department of Paediatric and Preventive Dentistry, Faculty of Medicine, University of Ljubljana, Ljubljana, Slovenia; 2 Institute of Preclinical Sciences, Veterinary Faculty, University of Ljubljana, Ljubljana, Slovenia; 3 Department of Dental Diseases and Endodontics, Faculty of Medicine, University of Ljubljana, Ljubljana, Slovenia; 4 Department of Materials and Metallurgy, Faculty of Natural Sciences and Engineering, University of Ljubljana, Ljubljana, Slovenia; 5 Small Animal Clinic, Veterinary Faculty, University of Ljubljana, Ljubljana, Slovenia

**Keywords:** Molar-root incisor malformation, Tooth development, Pulp biology, Histology, Scanning electron microscopy

## Abstract

**Background:**

Molar-root incisor malformation (MRIM) is a novel dental phenotype likely related to a patient’s past medical history. This case aimed to confirm MRIM by histological and scanning electron microscopy (SEM) examination for the first time in a patient diagnosed with autoimmune lymphoproliferative syndrome (ALPS) and to propose a possible link between ALPS and MRIM that could be attributable to abnormally proliferated bone marrow.

**Case presentation:**

A 12.5-year-old boy with an extensive medical history, including diagnosis of ALPS, was examined clinically and radiologically to elucidate the reason for pain primarily originating from the area of the lower left permanent first molar tooth (PFM; tooth 36). Dental examination and radiographic survey revealed abnormal pulp cavity morphology of all four PFMs, and these were extracted, resolving the dental pain in the patient. The extracted PFMs were subjected to light microscopy, SEM evaluation and mineral density and elemental composition analyses. Histology of two PFMs revealed the presence of dentin-, bone- and cartilage-like tissues with abundant blood vessels occupying the majority of the pulp chamber. The root canals were obliterated with mineralized structures resembling pulp stones. Two different, highly mineralized abnormal tissues filling the majority of the pulp chamber revealed by SEM and confirming the diagnosis of MRIM displayed a mineral density and elemental composition similar to those of enamel and dentin, respectively.

**Conclusions:**

It appears likely that in addition to the complex medical history during early childhood in the present case, extensive lymphoid infiltrates that are possible in ALPS patients can be regarded as a cofactor in the development of MRIM by exerting considerable pressure on the developing tooth bud and providing cells capable of differentiating into diverse cell types.

## Background

In 2014, a new type of dental malformation was described by Witt et al. [1], who found that root malformation of the permanent first molars (PFMs) was associated with a distinct structure: the ectopic mineralized plate or the cervical mineralized diaphragm (CMD). A comparable condition was described by Lee et al. [2], who termed this molar-incisor malformation (MIM) and described it as affecting PFMs and, in some cases, maxillary incisors. Another 30 similar cases were later described, and the condition was named molar root-incisor malformation (MRIM) [3]. An overview of previously published findings in teeth of patients with MRIM is presented in Table 1.

**Table 1:** Summary of the articles on the medical history and clinical characteristics of patients with MRIM

Author(s)	Patient (s)	Medical history	Dental features	
			PFMs	Other affected teeth and MRIM-related clinical data
Witt et al., 2014 [1]	8-year-old boy	At the age of 9 months, osteomyelitis of the left femur was successfully treated with antibiotics (cephalosporine, penicillin, lincosamide and glycopeptide).	In both cases, the roots of all four PFMs were malformed, with barely visible or very short roots and narrow appearance of the pulp cavities.	All PFMs extracted.
	10.5-year-old girl	Premature delivery (36th week) due to astrocytoma of the pregnant mother was reported. The mother was treated with corticosteroids and breastfed the newborn for two months. The girl had frequent middle ear infections from 2 years of age, which were treated with amoxicillin and clavulanic acid.		
Lee et al., 2014 [2]	*N* = 12 Male: 6 Female: 6 Aged 4–13 years	Ten of the patients had at age 1 to 2 years meningitis (3), brain injury by dystocia (1), hydrocephalus (1), spina bifida (1), cerebral cyst (1), cephalohematoma (1), or seizure (2).	In all patients, both mandibular PFMs were affected. Seven patients also had affected maxillary PFMs. Affected PFMs had thin, divergent or short roots and normal contour and surface strength of the crowns.	Mandibular deciduous second molars: 5 patients. Maxillary incisors (wedge-shaped defect at the cervical portion): 7 patients. Additional pathology reported: impaction of the PFMs, space loss due to early exfoliation of the deciduous second molar, impaction, hypo-occlusion, dental caries, adjacent tooth eruption disorder, periodontitis, spontaneous pain.
Lee et al., 2015 [5]	6-year-old girl	Premature birth (28th week, birth weight of 1.1 kg) was reported; at 8 weeks, she was diagnosed with perinatal asphyxia. At the age of 17 months, she was diagnosed with frontal intracerebral hemorrhage and a zygomaticomaxillary fracture.	All PFMs had barely developed roots, partly obliterated pulp cavities, constriction in the crown area, thickened pulpal floor, convex appearance of the furcation floor and normal tooth crown contour.	All PFMs extracted at the age of 9 during orthodontic treatment.
Wright et al., 2016 [3]	*N* = 30 Male: 18 Female: 12	During the neonatal period, patients had meningomyelocele or sacral dimple (7), meningitis (6), preterm birth (4), or chronic renal disease (4). In individual patients, the following were reported: meconium aspiration, urinary tract infections, hemiplegia (stroke), cerebral thrombosis, possible cerebrovascular accident, or cerebral palsy with placenta previa. No major problems were reported in 4 patients.	In all patients, all four PFMs were affected; dysplastic root formation and diminished pulp chamber of the PFMs were observed.	Deciduous second molars: - all four (14 patients) - both mandibular (one patient) Maxillary central incisor (12 patients) Maxillary and mandibular canine (5 patients) One patient had all PFMs, all permanent incisors and canines, and two premolars affected.
McCreedy et al., 2016 [6]	8-year-old boy	Sacrococcygeal teratoma was diagnosed prenatally and excised the second day after birth.	In both patients, all PFMs were present with abnormal morphology of the roots (hypoplastic and malformed) and narrow pulp canals but normal contour of the crowns.	Ectopic eruption of the mandibular PFMs. Ectopic eruption of the permanent maxillary second molars (one patient).
	9-year-old girl	Premature birth (28th week) was reported; at six months, she was diagnosed with asthma (treated with fluticasone propionate and albuterol).		
Yue and Kim, 2016 [4]	13-year-old-boy	A few days after birth, he was hospitalized for 10–12 weeks due to staphylococcus infection.	Mandibular PFMs had malformed roots (thin, narrow, short) and constricted pulp chambers.	Maxillary central incisors with a wedge-shaped defect at the cervical area.
Brusevold et al., 2017 [7]	*N* = 6 Aged 8–12 years	1. Born with brain blood clot; epileptic seizures until the age of 7 years 2. Abdominal tumor surgically removed at the age 3 months 3. Hydrocephalus, 3 ventricle shunts; hospitalized several times up to 1.5 years of age due to brain abscesses 4. Acute caesarean section, zygomatic cavernous hemangioma 5. Difficult delivery: tight nuchal cord, birth asphyxia, intracranial hemorrhage; cerebral palsy 6. Seizures and ischemic stroke the fifth day after birth	In all patients, all four PFMs were affected. Mandibular PFMs: deformed, twisted, narrow, tiny wedge-shaped roots or completely missing roots. Maxillary PFMs: underdeveloped malformed narrow roots.	Deciduous second molars: both mandibular and a right maxillary had no roots; maxillary left was missing (one patient). Maxillary permanent incisor with cervical constrictions (four teeth in two patients). Pain, abscess, fever and/or pus in conjunction with five mandibular PFMs.
Choi et al., 2017 [11]	6-year-old-girl	No history of systemic diseases or medical events at birth.	In both cases, roots of the mandibular PFMs were undeveloped, and roots of the maxillary PFMs were short and thin. In one case, roots of all PFMs were thin and convergent.	Deciduous second molars: one with a slit-shaped pulp cavity and atypical roots, other three missing (one patient). Maxillary permanent incisor with wedged-shape enamel defects on the crown (one patient). In the first case, the mandibular PFMs had to be extracted, and in the second case, two years after extraction of the mandibular PFMs, extraction of the maxillary PFMs had to be performed.
	9-year-old-girl	Premature birth (30th week, birth weight of 2.2 kg).		
	8-year-old-boy	Surgery for myelomeningocele immediately after birth.		

*N* - number of patients included

MRIM is characterized by underdeveloped aberrant roots of the PFMs with the crowns of these teeth having normal contour and surface strength [2]. Typically, the roots of all of the PFMs, especially those on the mandible, are affected [4]. The pulp chamber of the PFM is abnormal, being constricted into a narrow straight form in the crown [1]. In addition to the PFMs, the permanent maxillary incisors and/or primary second molars [2] or canines and mandibular incisors can be affected [3] (Table 1). Teeth with MRIM show diverse clinical problems [2, 3, 5–7], and endodontic treatment of such teeth is typically complicated [4, 6]. Hence, knowledge of pulp space morphology is essential for the development of a rational treatment plan [8]. Recommendations for diagnosis/treatment planning of MRIM were recently made by Brusevold et al. [7].

Tooth root malformations can occur as a result of various genetic and environmental factors (reviewed in [9]). Premature termination of root development can be due to infection, trauma, chemotherapy or radiation therapy [1]. Disruption in the development of many roots can be associated with dentine dysplasia type I and regional odontodysplasia [5]. In these cases, the root dysplasia is generalized or affects certain sections of a dental arch [1]. Molar root hypoplasia is observed in patients with Schimke immunoosseus dysplasia; its likelihood increases with the severity of the disease [10]. However, this autosomal-recessive disorder is also characterized by other dental anomalies (microdontia, hypodontia), dimorphic features (facial dimorphism, a short neck, hyperpigmented macules, protuberant trunk, short limbs), renal dysfunction, and T-cell immunodeficiency [10].

The etiology of MRIM remains unclear. A common feature reported in the majority of patients with MRIM is a serious medical condition and treatments (especially antibiotic) during the first 2 years of life [1–7]. No particular disease has been identified as a causal factor in MRIM [11] (Table 1). It appears that severe systemic conditions likely provoke a secondary effect that is expressed locally and affects the development of the tooth bud, resulting in the malformation of PFMs. Different environmental stressors occurring during early childhood have been associated with the abnormal formation of the MRIM tooth roots [3].

Autoimmune lymphoproliferative syndrome (ALPS) is an extremely rare disorder [12] characterized by the increased size of selected organs (mainly lymphadenopathy, hepatomegaly and splenomegaly) resulting from an abnormally large number of lymphocytes accumulating in the tissues as well as autoimmune destruction of blood cells (hemolytic anemia, thrombocytopenia and neutropenia) [13]. The bone marrow of ALPS patients is also commonly affected by lymphocytosis, with the possibility that extensive infiltrates can replace normal bone marrow elements [12]. In two-thirds of patients, a mutation in the *FAS* gene has been confirmed; in many cases, the etiology remains undefined [14].

Data on the oral health of ALPS patients are scarce. To the best of our knowledge, only one study, involving seven patients, has investigated the oral pathologies of patients with ALPS [15]. The authors of that study concluded that ALPS patients exhibit a wide spectrum of signs and symptoms affecting oral soft tissues (e.g., recurrent mucosal ulcers, a smooth tongue, gingivitis). Although caries was reported, no other dental diseases were mentioned, and no X-rays have been presented to date.

The aim of the present case report was to confirm MRIM in a patient diagnosed with ALPS by histological and scanning electron microscopy (SEM) examination and to determine the mineral density and elemental composition of the affected PFMs. To the best of the authors’ knowledge, no such case has been reported previously. Based on the findings, the authors propose a possible link between ALPS and MRIM that could be attributable to abnormally proliferated bone marrow.

## Case presentation

**Fig. 1:**
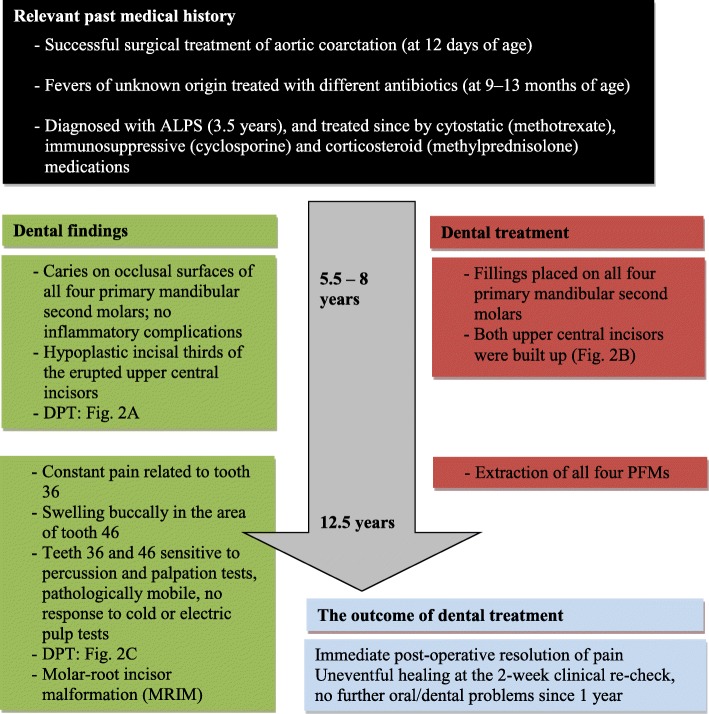
Timeline

**Fig. 2:**
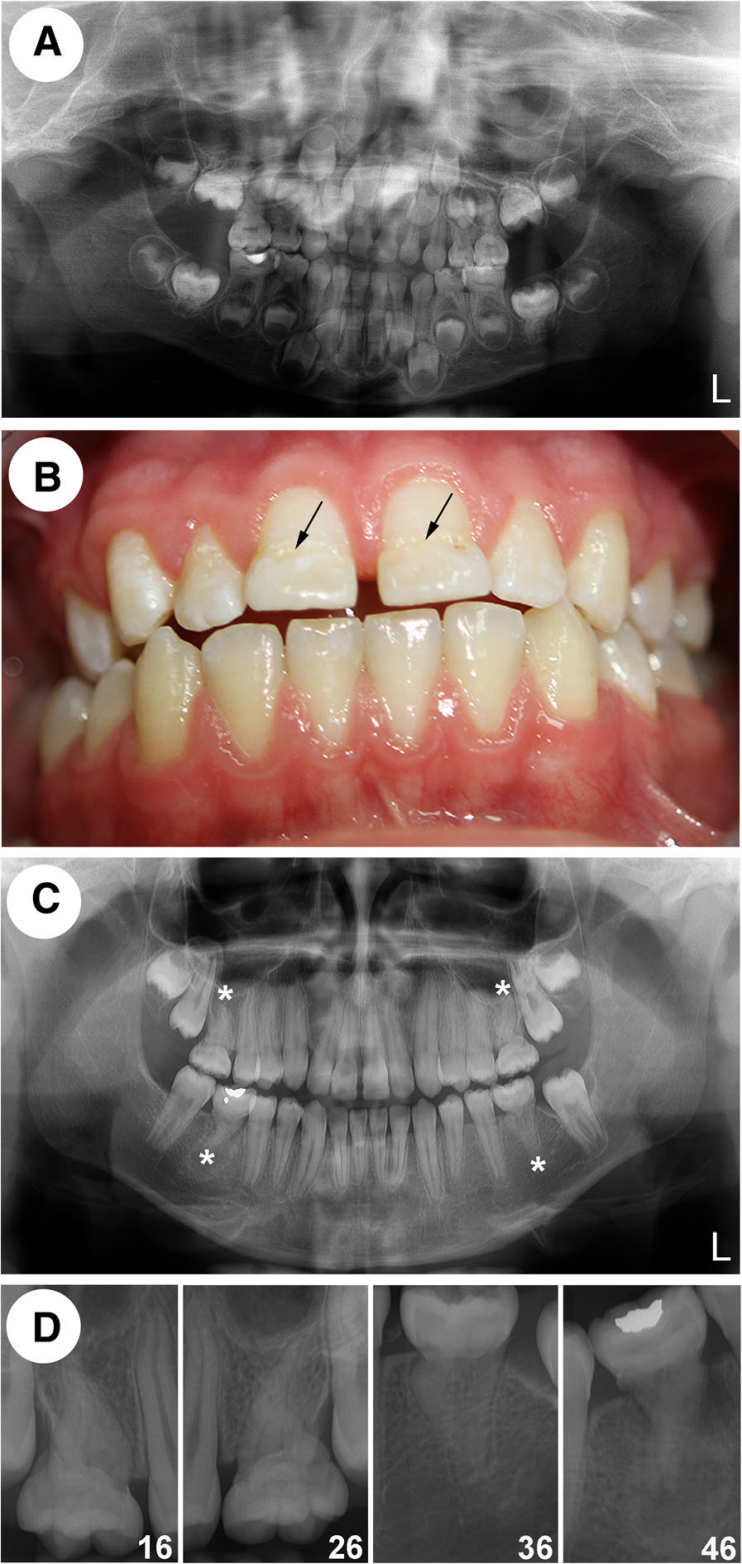
Radiographic and clinical characteristics of MRIM in a patient with ALPS. (**a**) A dental panoramic tomogram (DPT) taken when the patient was 6 years old reveals small pulp chambers and thin, short roots of all four primary second molars. In all four second primary molars, only thin horizontal lines can be seen presenting dental pulp chambers. The lack of dental pulp is more pronounced in both lower second primary molars. Comparison of the volumes of the dental pulps between the first and second primary molars reveals a substantial difference, with the second primary molars having a much smaller dental pulp chamber. Fillings are apparent on the primary mandibular second molars. Under the filling on the right side, there is a secondary caries lesion with no signs of periapical inflammation. (**b**) An intraoral image of the patient at the age of 12 years shows the permanent dentition. The tooth crowns show normal morphology except for the hypoplastic incisal thirds of the upper central incisors, which were both built up shortly upon eruption (marked with arrows). (**c**) A DPT taken at the same age (12 years) shows that in all four permanent first molars (PFMs), the pulp chambers are hardly recognizable and appear to be constricted into a narrow straight form and that the roots of these teeth are shorter and thinner than normal. (**d**) Periapical radiographs reveal barely visible contours of the dental pulp of all four PFMs. In particular, the lower PFMs show severely aberrant roots with no identifiable pulp chambers. In tooth 46, periodontal inflammation is visible, most likely a sequel of the aberrant tooth root

The medical history of a 12.5-year-old boy, referred due to pain in the area of the lower left PFM (tooth 36), reported serious health conditions since the first year of life (Fig. 1). At the age of 3.5 years, he was diagnosed with ALPS; although all of the findings were indicative of ALPS, there was no history of ALPS in the family and no mutation of the most commonly involved genes (*FAS, FASLG*) [13], as confirmed by genetic analysis. His dental history reported fillings on all second primary molars; however, no inflammatory complications were reported. A dental panoramic tomogram (DPT), obtained when the patient was 6 years old, is presented in Fig. 2a.

At the age of 12.5 years, a dental clinical examination revealed complete permanent dentition, and both upper central incisors were built up (Fig. 2b). Reportedly, this treatment was performed by a general dentist as soon as the incisors erupted as they had hypoplastic incisal thirds. Otherwise, the crown morphology was normal. On the cervical halves of the PFMs, poor mineralization of the enamel was identified, which most likely occurred during enamel formation. The remaining tooth crowns appeared intact. Except in the area of the right mandibular PFM, the oral mucosa was of normal coral pink color, size and resilience and showed no inflammatory or other pathologic signs. Swelling was observed buccally in the area of the right PFM (tooth 46), whereas the patient reported constantly present spontaneous pain related to the left PFM (tooth 36). Both mandibular PFMs were sensitive to percussion and did not respond to cold or an electric pulp test. The right one was pathologically mobile. Diagnostic evaluation findings are presented in Fig. 1. DPT and periapical radiographs revealed profoundly malformed pulp cavities and tooth roots of all four PFMs (Figs. 2c, d). There was an appreciable peri- and para-apical radiolucency related to tooth 46. In addition, DPT showed the presence of both maxillary third molars but no mandibular third molars.

Based on clinical and radiographic findings, a diagnosis of symptomatic apical periodontitis associated with the necrotic mandibular left PFM and of pulpal necrosis with acute apical abscess for the mandibular right PFM was reached. Due to the very poor prognosis for the endodontic treatment of mandibular PFMs with aberrant root canal morphology, the recommendation of the interdisciplinary team (endodontist, orthodontist and pediatric dentist) was extraction of all four abnormally formed PFMs. This was performed followed by an orthodontic space closure. Prior to any procedures, written informed consent was obtained from the patient and his parents. Extraction resulted in an immediate post-operative resolution of pain, and uneventful healing was observed at the 2-week clinical recheck. No further oral/dental problems have been reported.

### Histological analysis

**Fig. 3:**
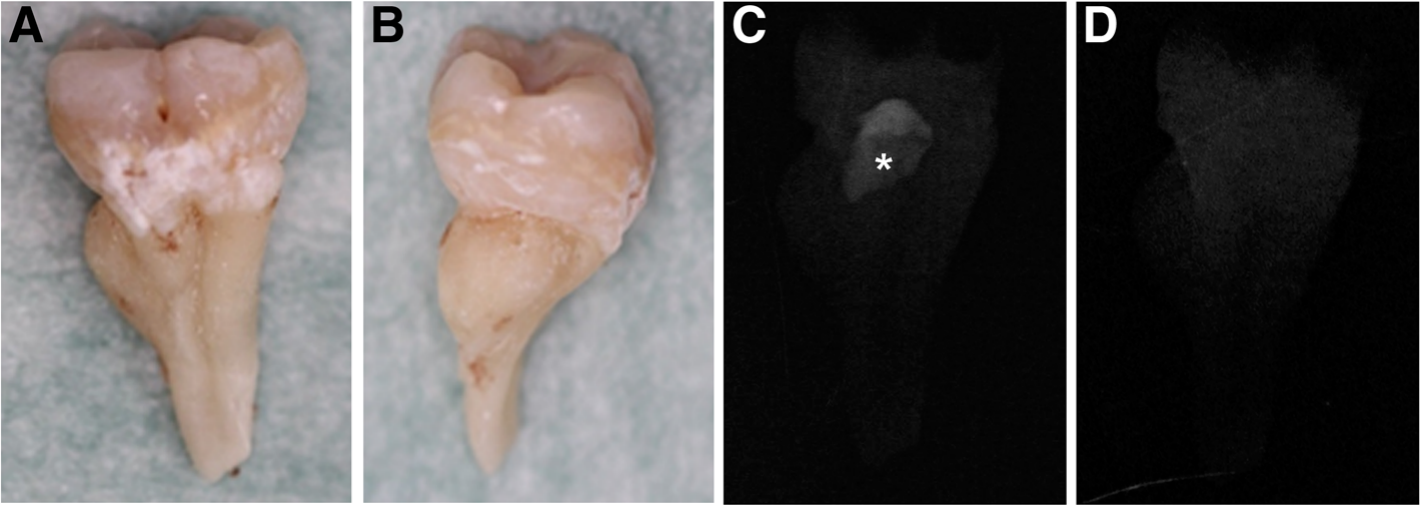
Photographs of the left mandibular first molar (36) taken before the demineralization procedure and progression of the demineralization process as monitored by dental radiographs. Tooth 36 from the (**a**) buccal and (**b**) mesial surface. Note the aberrant root morphology and a ribbon of hypomineralized enamel extending circularly around the tooth crown and toward the cervical area. (**c**) Radiograph of tooth 36 taken 14 weeks after demineralization with the mild bone-decalcification solution Osteosoft®. Note incomplete decalcification with unusual mineralized tissue in the area of the pulp chamber (asterisk). (**d**) The process was successfully completed after additional overnight decalcification with a 1:1 mixture of Osteosoft® and Osteomoll® solution

Teeth 16 and 36 (Figs. 3a, b) were fixed with 10% neutral buffered formalin and demineralized with the Shandon TBD-2 Decalcifier (Thermo Fisher Scientific Inc., Waltham, MA, USA) and the mild bone-decalcification solution Osteosoft® (Merck KGaA, Darmstadt, Germany), respectively [16, 17]. Progression of the demineralization was monitored by dental radiographs (Figs. 3c, d).

Both decalcified specimens were dehydrated and embedded in paraffin to prepare a series of 5-μm histological sections cut in a mesiodistal direction at 50-μm intervals using a Leica SM 2000R microtome (Leica Biosystems, Nußloch, Germany). The specimens were stained with hematoxylin and eosin (HE), a Masson-Goldner kit (Merck KGaA, Darmstadt, Germany), toluidine blue (pH 7.2) and alcian blue (pH 2.5; Merck KGaA, Darmstadt, Germany). A Nikon Microphot-FXA microscope equipped with a DS-Fi1 camera and NIS-Elements imaging software (NIS Elements D.32; Nikon Instruments Europe B.V., Badhoevedorp, the Netherlands) were used for histological examination. Representative tissue sections were presented using *Adobe Creative Cloud*.

**Fig. 4:**
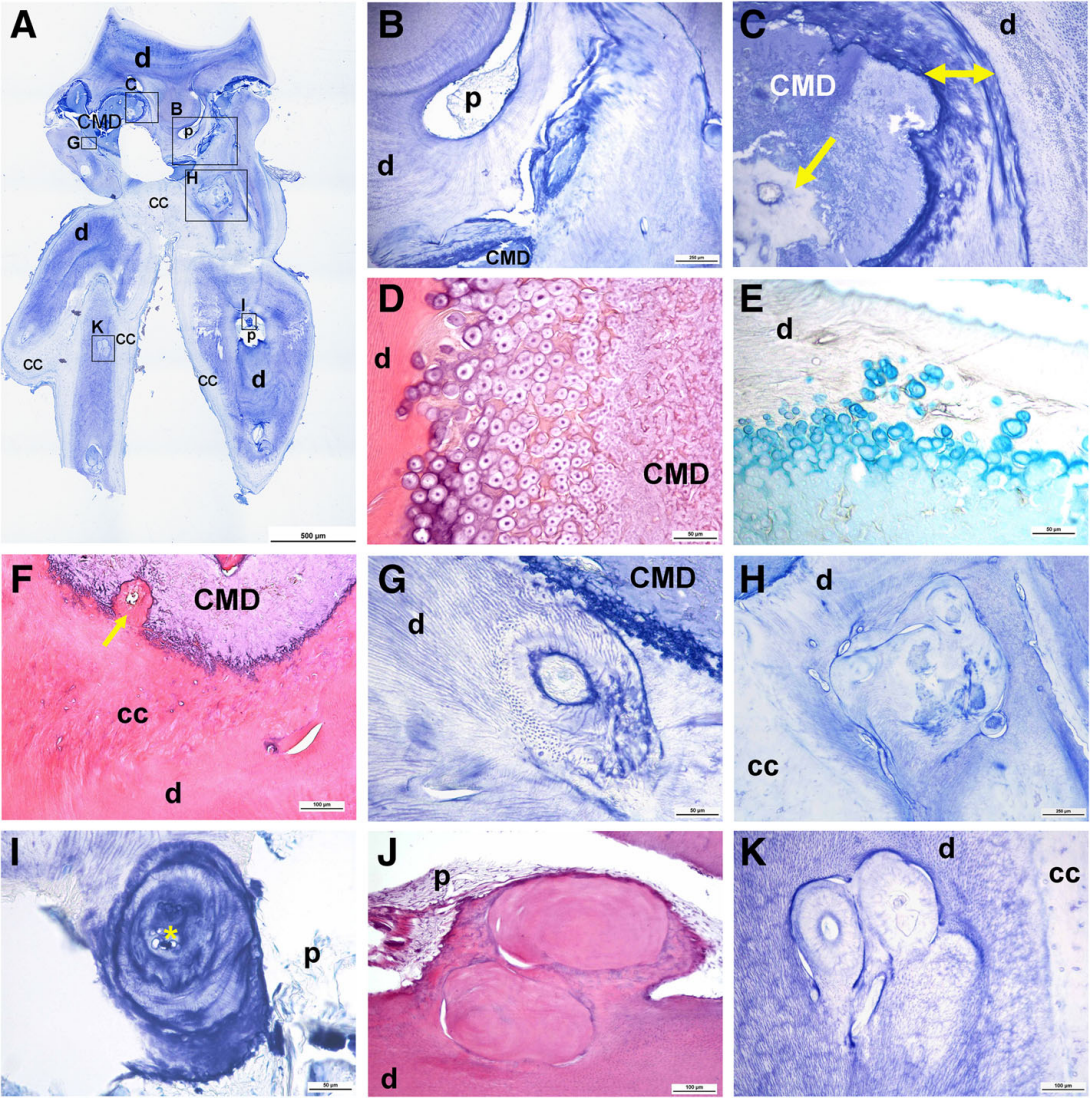
Histological characteristics of the right maxillary first molar (16). (**a**) An overview micrograph of the longitudinal section of the tooth shows a discontinuous cervical mineralized diaphragm (CMD) in the area of the pulp chamber and a narrow coronal pulp positioned above and interspersed between the regions occupied by the CMD. A smaller area occupied by radicular pulp is also apparent. Higher-magnification micrographs marked with rectangles (**b**) show the course of the dentinal tubules in the coronal dentin interspersed between the CMD, (**c**) amorphous tissue at the border between the CMD and dentin (yellow double-sided arrow) and the connective tissue canal containing blood vessels (yellow arrow). This connective tissue exhibits pale staining with toluidine blue, whereas the surrounding tissue consists of globules and a toluidine blue-positive interglobular matrix. (**d**) CMD consisting of globules and interglobular matrix. At the border with dentin (**e**), there are enlarged chondrocyte-like cells residing in the lacunae and surrounded by the alcian blue-positive matrix, suggestive of cartilage proteoglycans. (**f**) Tissue below the CMD resembles cellular cementum; note the ingrowth of the connective tissue canal containing blood vessels (yellow arrow). (**g**) Single or (**h**) multiple denticle-like structures are present below the CMD and (I) within the root canal. Denticle-like structures are either (**j**) partly or (**k**) completely incorporated into the dentin. Cells present within the central area (asterisk) are suggestive of an immature denticle (**g**). A layer of columnar odontoblasts that should surround the outer surface of intrapulpal stones is not obvious. cc: cellular cementum; CMD: cervical mineralized diaphragm; d: dentin; p: pulp. (**a**, **b**, **c**, **g**, **h**, **i** and **k**): toluidine blue (pH 7.2); (**d**, **f**, **j**): HE; (**e**): alcian blue (pH 2.5)

**Fig. 5:**
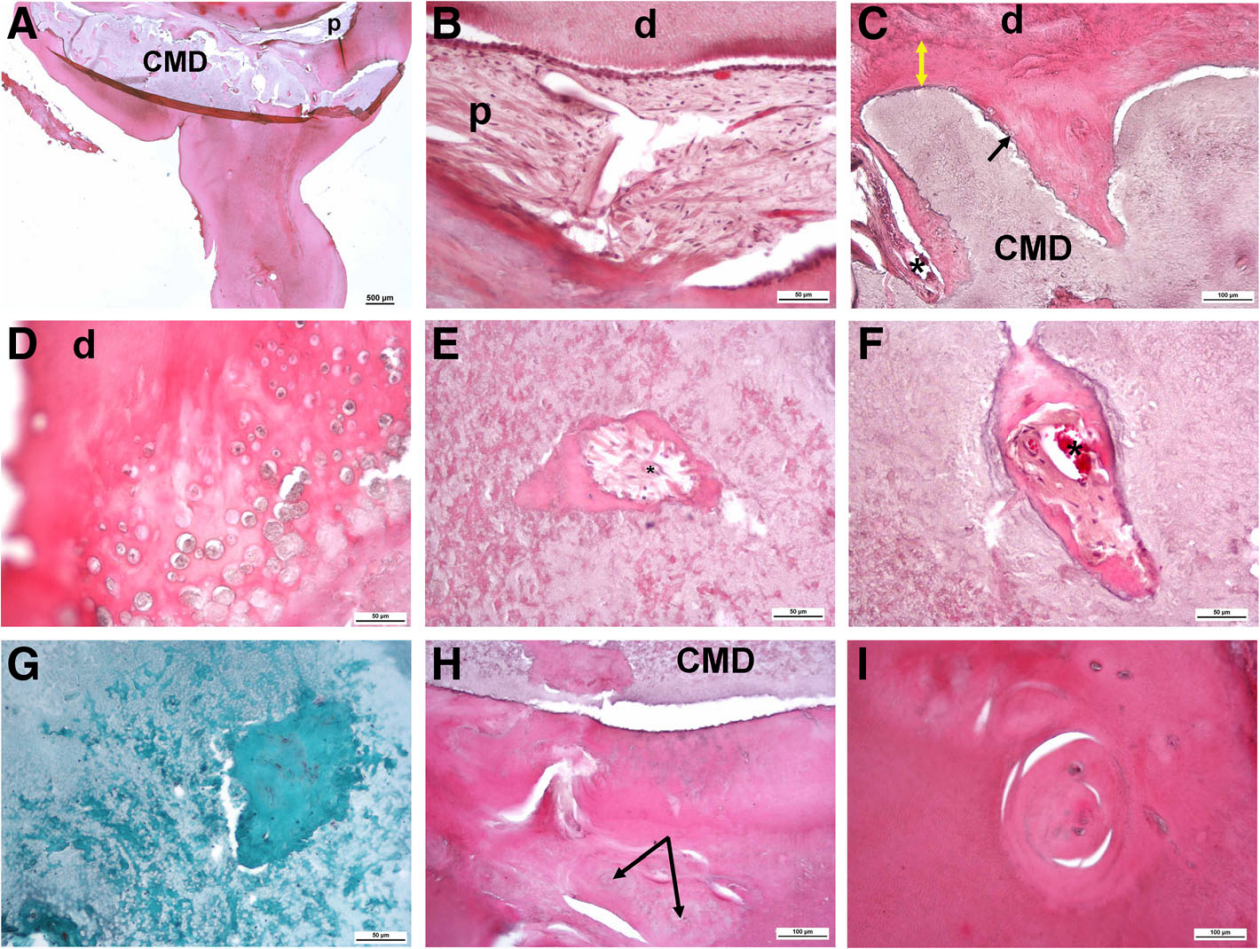
Histological characteristics of the left mandibular first molar tooth (36). (**a**) An overview micrograph of the longitudinal section of the tooth shows that the pulp chamber is almost completely obliterated by the CMD. Note remnants of the pulp located above the CMD and a root-like structure projecting from the cervical area. Higher-magnification micrographs (**b**) show the fibrous appearance of the coronal pulp tissue and (**c**) amorphous tissue resembling tertiary dentin at the pulp periphery, i.e., at the border between the CMD and occlusal dentin (yellow double-sided arrow), with individual cells residing in the lacunae resembling chondrocytes (black arrow). (**d**) Numerous chondrocyte-like cells are residing in the thin dentinal wall. In the heterogeneous structure of the CMD, note (**e**, **f**) connective tissue canals containing blood vessels (asterisks), (**g**) green-stained collagen fibers; below the CMD, note (**h**) abnormal dentin and tissue resembling cellular cementum, with connective tissue canals and round-to-ovoid structures (arrows) of concentrically arranged collagen fibers and locked-in cells. (**i**) Round-to-ovoid structures are also present in the root-like extension. CMD: cervical mineralized diaphragm; d: dentin; p: pulp. (**a**-**f** and **h**-**i**): HE; (**g**): Masson-Goldner staining

Examination by light microscopy revealed profoundly folded dentin (especially in the root portion) as well as an unusual tissue filling in the majority of the pulp chamber, comparable to CMD [1]. Only minor areas of preserved normal pulp were evident (tooth 16; Fig. 4a, b; tooth 36; Fig. 5a, b). The course of the dentinal tubules appeared normal only in the occlusal third of the crown (Figs. 4a, b). The CMD contained connective tissue canals with blood vessels, many of which resembled osteons (Figs. 4c, f and 5e, f). The surrounding tissue consisted mainly of globules and interglobular matrix that contained only scarce collagen fibers (Figs. 4d and 5g). At the border between the CMD and the occlusal dentin, amorphous tissue resembling tertiary dentin was present (Figs. 4c and 5c). In areas where the dentinal wall was thinner, there were chondrocyte-like cells (Figs. 4d, e). The cervical area (where the floor of the dental pulp chamber and furcation should have been) contained cellular cementum and some periodontal ligament tissue. The root canals were obliterated with mineralized structures resembling pulp stones (Figs. 4g-k) with remnants of normal pulp tissue (Figs. 4a, b, i, j). Sequential histological sections cut in a mesiodistal direction at 50 μm displayed similar findings.

### Scanning electron microscopy

Tooth 46 was fixed in 10% neutral buffered formalin, rinsed, bisected bucco-palatally and embedded in epoxy resin (Araldite, Ciba-Geigy, East Lansing, MI, USA) with the cut side exposed. After polymerization, the exposed axial cross-section was polished, etched with 37% orthophosphoric acid for 30 s, rinsed with distilled water spray for 30 s, dried with compressed air, dehydrated with 70% ethanol, dried again and sputter-coated with carbon (Vacuum Evaporator, Type JEE-SS; Japan Electron Optics, Tokyo, Japan). Subsequently, the sample was subjected to ultrastructural analysis via SEM (JEOL JSM - 5610, JEOL, Tokyo, Japan) performed in the secondary electron imaging (SEI) and backscattered electron (BSE) modes. Micrographs were recorded at 15 kV and a working distance of 20 mm.

**Fig. 6:**
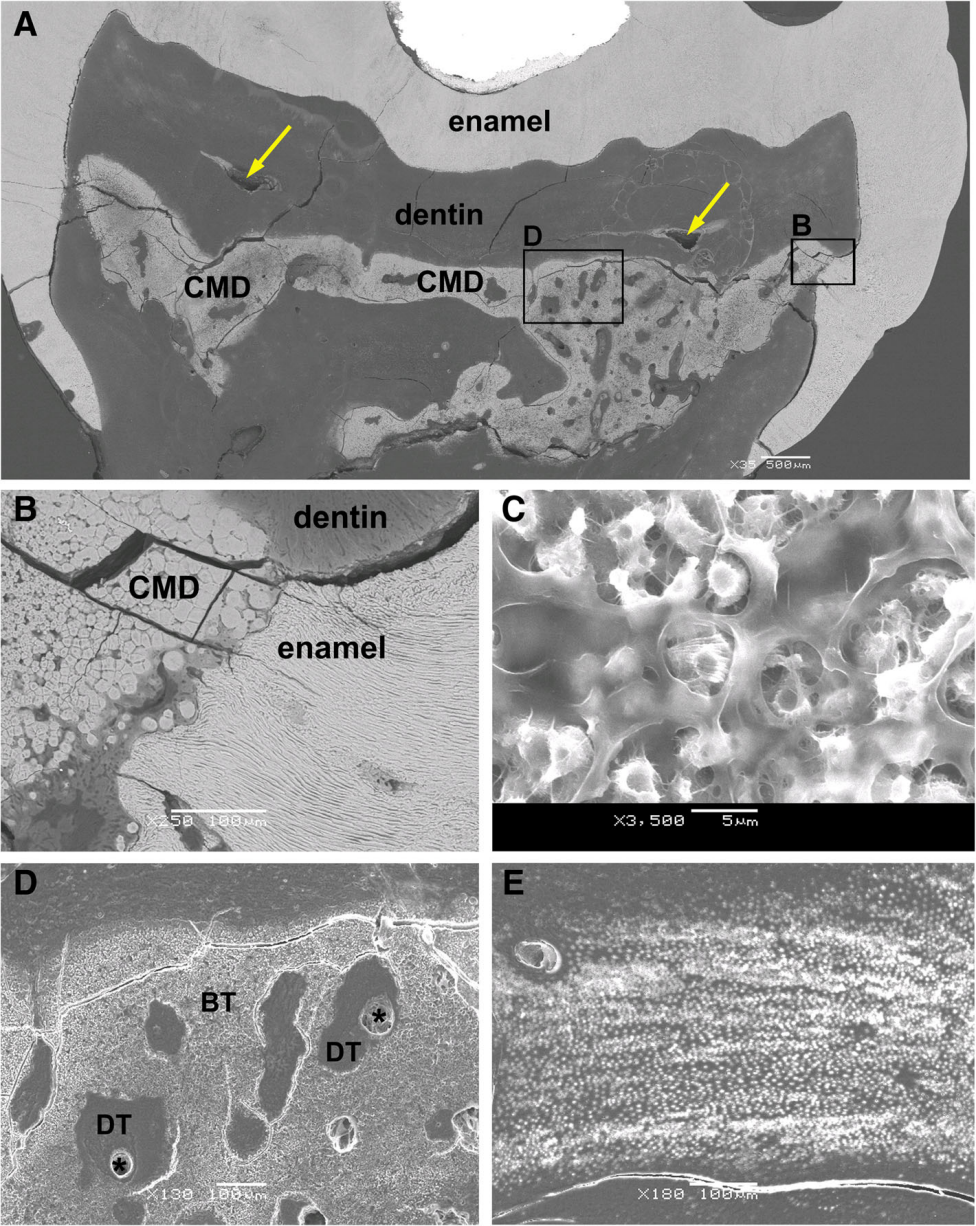
Scanning electron microscopy (SEM) micrographs of the right mandibular first molar tooth (46). (**a**) A transversely cut crown of tooth 46 reveals that no normal pulp chamber is present except for the two empty spaces, which indicate areas where dental pulp used to be located (marked with arrows). The central part of the tooth crown is filled with an ectopic mineralized tissue of brighter and darker appearance, i.e., a CMD (× 35, SEI). On the right side, at approximately half of the height of the tooth crown, only a thin layer of aberrant dentin delimits the mineralized content of the pulp chamber from the enamel (area in rectangle B). (**b**) Under higher magnification, nearly direct contact between the CMD and enamel can be observed; spherical structures comprising brighter areas of the CMD are in contact with either enamel or bordering dentin (× 250, BSE). (**c**) Brighter tissue of the CMD consists of spherical structures resembling cells, with protruding cytoplasmic processes, surrounded by amorphous extracellular matrix (× 3500, SEI). (**d**) The “darker tissue” of the CMD is located between the “brighter tissue”; in these parts, blood vessels (asterisk) are visible and surrounded by dentin-like tissue (area in rectangle D; × 130, SEI). (**e**) Blood vessels are also found in the dentin, which is visible in the central part of the composite image (**a**) and is surrounded by the CMD in the area where pulp tissue is normally present (× 180, SEI). BT: “brighter tissue”; CMD: cervical mineralized diaphragm; DT: “darker tissue”

The SEM images revealed a normal structure and thickness of the enamel, areas with normal and abnormal dentin, and an almost completely mineralized pulp chamber (i.e., a CMD). This CMD was composed of brighter and darker areas (Fig. 6), referred to as “brighter tissue” and “darker tissue” in the text below, corresponding to globules/interglobular matrix and connective tissue canals with blood vessels, respectively (cf. Figs. 4 and 5).

### Mineral density and elemental composition analyses

Mineral densities and the elemental composition of four areas were compared: 1) brighter tissue and 2) darker tissue of the abnormal hard tissue located at the site of the pulp chamber (CMD), 3) dentin and 4) enamel.

For mineral density analysis, 10 BSE images of each selected tissue were taken at a magnification of 1500x, 15 kV and a working distance of 20 mm. For enamel, the images were taken approximately in the middle of the whole enamel thickness, on the occlusal and proximal sides, at the occlusal half of the tooth crown. For dentin, images were obtained from normal dentin located on occlusal side between the enamel and pulp chamber area, approximately in the middle of its thickness. For brighter tissue and darker tissue of the CMD, we randomly took images in the area of the CMD in regions where we found typical appearance of brighter and darker tissue. Using the OpenCV library in Python (Windows, Microsoft), for each pixel in an image, the grayscale value in the range of 0 to 255 (black to white) was determined, and the average grayscale value was then computed for each image.

For elemental analysis, five representative images were taken of each of the four tissues, selected in the same manner as described above. Elemental analysis was performed with energy dispersive X-ray spectroscopy-EDXS (500 Digital Processing; IXRF Systems, Houston, TX, USA) at a working distance of 20 mm, an acceleration voltage of 15 kV and a counting time of 80 s. This approach was conducted on the entire surface area of the SEI images under 1500x magnification. For each of the four tissue groups, five images were analyzed, and the values obtained were expressed as the mean ± SD. The results of the EDXS spectra were further evaluated in relation to the carbon-oxygen ratio (C:O) and calcium-phosphorous ratio (Ca:P) as described previously [18].

The statistical significance of differences between analyzed tissues was determined by one-way analysis of variance (ANOVA) followed by Tukey’s post hoc test using SPSS 20.0 for Windows (SPSS Inc., Chicago, Ill, USA). A *P*-value≤0.05 was considered statistically significant.

**Fig. 7:**
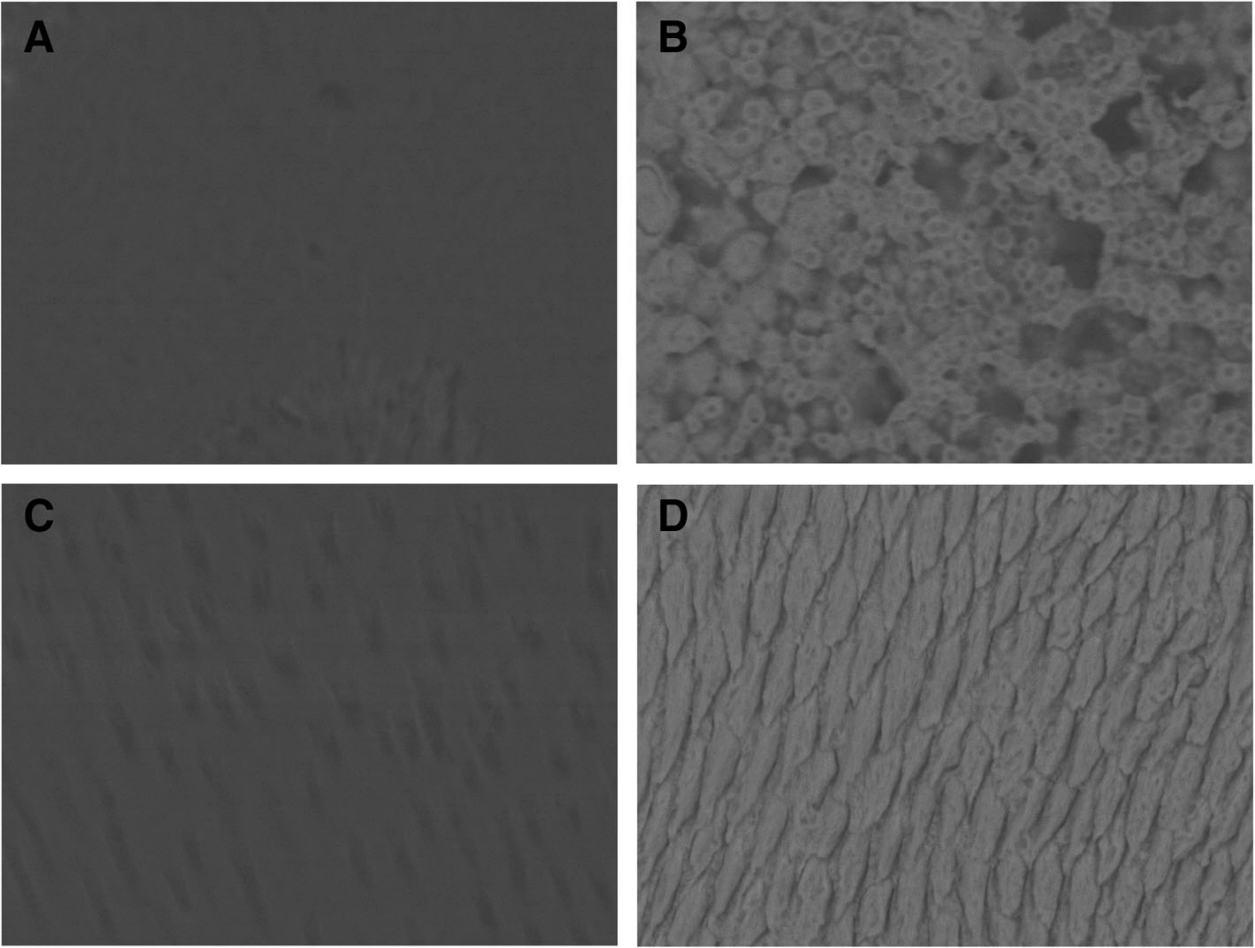
Mineral density of different dental tissues. Representative SEM-BSE micrographs of (**a**) “brighter tissue” and (**b**) “darker tissue” of the CMD, (**c**) dentin and (**d**) enamel (× 1500, BSE)

Mineral density and elemental composition analyses confirmed the similarities between the brighter tissue and enamel, and between the darker tissue and dentin. Figure 7 (a-d) shows representative SEM images of each of the tissues from the crown of the tooth. The mineral density of the darker tissue was comparable to that of the dentin (29.16 ± 0.37 and 29.24 ± 0.16, respectively; *P* = 0.458), whereas the mineral density of the brighter tissue was lower than that of the enamel (39.64 ± 0.88 and 42.63 ± 0.49, respectively; *P* = 0.05). Data on the elemental composition are summarized in Table 2. The darker tissue and brighter tissue displayed elemental compositions that resembled dentin and enamel, respectively. The C:O ratio differed significantly between the pairs of groups (enamel/brighter tissue vs. dentin/darker tissue), whereas the Ca:P ratio was only reduced in the darker tissue (Table 2).

**Table 2:** Elemental composition of different tooth tissues. Values are the mean ± SD. The number of images used in each analysis is given in parentheses

Parameter	Darker area (5)	Brighter area (5)	Dentin (5)	Enamel (5)
Carbon (%)	78.81 ± 3.10^b,d^	29.60 ± 1.39^a,c,d^	79.31 ± 1.76^b,d^	14.21 ± 0.95^a,b,c^
Oxygen (%)	10.94 ± 2.42^b,d^	16.75 ± 0.26^a,c^	10.14 ± 0.61^b,d^	18.75 ± 0.95^a,c^
Phosphorus (%)	3.77 ± 1.01^b,d^	18.98 ± 0.30^a,c,d^	4.32 ± 0.88^b,d^	23.77 ± 0.47^a,b,c^
Calcium (%)	5.25 ± 1.60^b,d^	33.36 ± 1.39^a,c,d^	6.49 ± 1.69^b,d^	42.56 ± 0.84^a,b,c^
C:O ratio (%)	7.49 ± 1.61^b,d^	1.77 ± 0.10^a,c^	7.74 ± 0.32^b,d^	0.76 ± 0.06^a,c^
Ca:P ratio	1.42 ± 0.32^b,d^	1.76 ± 0.08^a^	1.49 ± 0.14	1.79 ± 0.04^a^

The statistical significance of differences between tooth tissues was analyzed by one-way ANOVA followed by Tukey’s post hoc test

^a^significantly different from the darker area

^b^significantly different from the brighter area

^c^significantly different from the dentin

^d^significantly different from the enamel

Differences were considered significant at *P* ≤ 0.05

## Discussion and conclusions

This report describes MRIM in a young boy affected by ALPS, with clinical and radiographic findings in agreement with those of previous reports [1–3, 5–7]. Mandibular PFMs with MRIM caused spontaneous pain, and an apical abscess in the absence of dental caries was observed. Abscesses in these teeth are thought to be caused by pulp necrosis induced by pulp chamber obliteration or by pulp exposure induced by dentinal defects [5]. Endodontic treatment of the MRIM tooth with a profoundly calcified pulpal floor and malformed root is very difficult or unfeasible [4], which is why the treatment option to extract these teeth was chosen.

The histological findings were largely consistent with those previously reported for MRIM-affected teeth [1, 3, 5]. Obscure mineralized content filling in the majority of the pulp chamber (i.e., CMD) consisted of several different tissues, including enamel-like tissue as determined by mineral density and elemental composition analyses. Specifically, when mineral density and elemental composition were analyzed, the darker tissue of the CMD resembled dentin, whereas the brighter tissue of the CMD was more enamel-like. Witt and coworkers [1] reported similar mineral densities of the “interglobular part of CMD” and dentin and a much higher mean density value of the “globular part of CMD”, which approached the mean value of the enamel. Three distinct layers of tissues reportedly visualized by microcomputed tomography in the pulpal floor of MRIM-affected teeth [5] were not distinguished in our case. The root canal was almost completely filled in by structures resembling pulp stones, similar to previous reports [1, 3]. In MRIM, the exact cause of pulp stone formation is not known but may be related to systemic disease [19].

The formation of MRIM has been proposed to result from damage to the vascular plexus at the base of the dental papilla during crown development, giving rise to the precipitation of calcified globules, with the interglobular components of the CMD deriving from the dental follicle [1]. Alternatively, the middle layer of the CMD may originate primarily from the apical pulp and partially from the dental follicle, with the lower layer formed by the dental follicle [5]. However, these hypotheses cannot explain the origin of the enamel-like tissue in the mineralized content of the pulp chamber. Its presence may be the result of Hertwig’s epithelial root sheath (HERS) cell differentiation into ameloblasts [20] or differentiation of other pluripotent stem cells into ameloblast-like cells in the presence of HERS cells [21]. Alternatively, bone marrow-derived cells, which appear to have the greatest capability to differentiate into diverse cell types [22, 23], including ameloblast-like cells [24], could contribute to the source of cells forming the CMD. Moreover, highly active bone marrow may represent a locally expressed factor as a secondary effect of a severe systemic condition, resulting in MRIM. This could also explain why PFMs (especially mandibular PFMs) are consistently affected by MRIM, whereas the occurrence of MRIM in other teeth varies. In a healthy newborn and in infants up to 3 years of age, hematopoietic marrow is distributed in both jaws, particularly in the mandible [25]. Before the age of one year, 100, 67 and 50% of subjects have hematopoietic marrow throughout the condyle, ramus and angle of the mandible, respectively [25]. With increasing age, hematopoietic marrow is gradually replaced with fatty marrow; in the most distal part of the mandible, conversion occurs by the age of 3 years [25]. If there was profoundly abnormal growth of bone marrow in the jaw, it would most likely be present in the area of developing mandibular PFMs. Accordingly, aberrant roots (divergent, twined, hypoplastic or undeveloped) and pulp chambers (constricted into a narrow straight form) of the PFMs are pathognomonic for MRIM. As extensive lymphoid infiltrates in the bone marrow are possible in ALPS patients [12], hypothetically, such proliferated bone marrow could exert pressure and present a mechanical obstacle that interferes with the developing tooth during the first year and thus plays a role in the etiology of MRIM. In support of this view, i) the aberrant pulp chamber and the abnormal course of the dentinal tubules indicate possible mechanical obstruction to the developing tooth bud, and ii) bone marrow-derived cells have the greatest capability to give rise to the diverse types of tissue found in the mineralized content of the pulp chamber area. Interestingly, the hearing loss observed in a murine model of ALPS (i.e., MRL/*lpr* mice) also results from defects in the bone marrow [26].

In summary, based on the rarity of ALPS and the lack of reports on oral/dental health in ALPS patients, it cannot be concluded that ALPS causes MRIM. At this point, the presented case may represent a unique, singular case, and one can only speculate about the MRIM incidence in ALPS patients. In addition, the environmental factors, medical conditions and medications to which the patient was subjected to during his first 2 years of life cannot be ruled out as possible cofactors in the development of MRIM in the presented case, but lymphoid infiltrates in the bone marrow, which interfere with the development of the tooth bud, should be considered as another possible (co)factor. Further investigations into the bone marrow abnormalities that serve as a link between ALPS and MRIM are warranted.

## Abbreviations

ALPS: Autoimmune lymphoproliferative syndrome
C: Carbon
Ca: Calcium
CMD: Cervical mineralized diaphragm
DPT: Dental panoramic tomogram
HERS: Hertwig’s epithelial root sheath
MIM: Molar-incisor malformation
MRIM: Molar-root incisor malformation
O: Oxygen
P: Phosphorus
PFM: Permanent first molar tooth
SEM: Scanning electron microscopy
